# Alterations in Bone and Erythropoiesis in Hemolytic Anemia: Comparative Study in Bled, Phenylhydrazine-Treated and *Plasmodium*-Infected Mice

**DOI:** 10.1371/journal.pone.0046101

**Published:** 2012-09-28

**Authors:** Robert Moreau, Diane Tshikudi Malu, Mathieu Dumais, Esther Dalko, Véronique Gaudreault, Hugo Roméro, Corine Martineau, Olha Kevorkova, Jaime Sanchez Dardon, Erin Lynn Dodd, David Scott Bohle, Tatiana Scorza

**Affiliations:** 1 Department of Biological Sciences, Université du Québec à Montreal, Montréal, Quebec, Canada; 2 Department of Chemistry, McGill University, Montreal, Quebec, Canada; University of Sydney, Australia

## Abstract

Sustained erythropoiesis and concurrent bone marrow hyperplasia are proposed to be responsible for low bone mass density (BMD) in chronic hemolytic pathologies. As impaired erythropoiesis is also frequent in these conditions, we hypothesized that free heme may alter marrow and bone physiology in these disorders. Bone status and bone marrow erythropoiesis were studied in mice with hemolytic anemia (HA) induced by phenylhydrazine (PHZ) or *Plasmodium* infection and in bled mice. All treatments resulted in lower hemoglobin concentrations, enhanced erythropoiesis in the spleen and reticulocytosis. The anemia was severe in mice with acute hemolysis, which also had elevated levels of free heme and ROS. No major changes in cellularity and erythroid cell numbers occurred in the bone marrow of bled mice, which generated higher numbers of erythroid blast forming units (BFU-E) in response to erythropoietin. In contrast, low numbers of bone marrow erythroid precursors and BFU-E and low concentrations of bone remodelling markers were measured in mice with HA, which also had blunted osteoclastogenesis, in opposition to its enhancement in bled mice. The alterations in bone metabolism were accompanied by reduced trabecular bone volume, enhanced trabecular spacing and lower trabecular numbers in mice with HA. Taken together our data suggests that hemolysis exerts distinct effects to bleeding in the marrow and bone and may contribute to osteoporosis through a mechanism independent of the erythropoietic stress.

## Introduction

Low bone mass density (BMD) and fractures are frequent in patients suffering from hemolytic hemoglobinopathies but their etiology is unknown. It is hypothesized that overexertion of hematopoiesis provokes bone marrow hyperplasia and reduces the amount of bone generated [1], causing bone distortion/fragility and enhanced bone resorption [2]. This hypothesis is sustained by the fact that low BMD is found in mice bled chronically for 10 months or in total-body irradiated mice [3]. High-resolution 3D studies locate erythropoietic islands in the inter-trabecular spaces of the bone marrow [4] and modifications in the vascular structure, in the proportion of hematopoietic lineages and in the remodeling of the trabecular bone are expected to assure replenishment of erythrocytes in response to chronic erythropoietic stress. These hemolytic conditions are also characterized by dysfunctional bone marrow erythropoiesis [5], [6], [7] and extramedular erythropoiesis [8], [9], [10].

The chronic hemolysis common to hemoglobinopathies as thalassemia and sickle cell disease is concurrent to increased oxidative insults in red blood cell (RBC), resulting from genetically defective hemoglobins 1,11,12,13. Under homeostatic conditions, hemoglobin released in hemolysis is rapidly scavenged by haptoglobin and internalized by macrophages, which degrade it through the heme oxygenase-1 (HO-1) system [14]. Extracellular hemoglobin rapidly oxidizes to methemoglobin and releases prosthetic heme groups that are toxic and pro-inflammatory [15] and plasma hemopexin and albumin greatly limit the deleterious effects of heme through their tight binding [16]. However, an excessive release of hemoglobin drastically decreases haptoglobin levels, resulting in accumulation of free heme and generation of reactive oxygen species (ROS), which through a positive feedback loop generate more methemoglobin and free heme. The fact that pathologies as β-thalassemia [17] and sickle cell disease [18] are characterized by high concentrations of heme confirm physiological exhaustion of hemoglobin and heme scavengers. Free heme is highly lipophilic, crossing cell membranes and various lipid structures and provoking oxidative stress through its redox activity [19].

The maintenance of adequate BMD relies on the balance between resorption and formation processes in bones (reviewed in [20]). Bone forming osteoblasts secrete matrix components such as collagen type I as well as non-collagenous protein (i.e. osteocalcin, OCN) and promote subsequent matrix mineralization. Following a period of activity, osteoblasts enter apoptosis, terminally differentiate to osteocytes surrounded by bone matrix or remain on the bone surface as quiescent bone-lining cells. Osteoblasts also provide factors essential for the differentiation of osteoclasts, such as macrophage colony stimulating factor (M-CSF) and the cell surface receptor activator of NF-κB ligand (RANKL) which binds to its receptor RANK expressed on osteoclast progenitors, and thereby induces the fusion of mononuclear monocyte precursors into multinucleate osteoclasts and activated osteoclasts secrete tartrate-resistant acid phosphatase 5b (TRAP5b). Since osteoblasts also release osteoprotegerin (OPG) which acts as a decoy receptor for RANK and inhibits osteoclast differentiation, osteoblasts tightly regulate the levels of bone resorption through the OPG/RANKL ratio [21], playing a central role in bone formation and preserving the bone remodeling delicate balance [22].

Inflammation is also a known modulator of bone function [23], [24], [25] and heme-dependent induction of anti-inflammatory HO-1 has been reported to block the maturation and activity of osteoblasts in a study [26], whereas an opposite stimulatory effect has been revealed by an independent research group [27]. Since heme-dependent inhibition of osteoclastogenesis and osteoclast activity *in vitro* and *in vivo* has also been described [28], heme may contribute to dysfunctions of bone cells which account for low BMD in hemolytic conditions.

Herein, the *Plasmodium chabaudi adami* (DK) (rodent malaria) and the phenylhydrazine (PHZ) models were used to characterize the effect of short-term hemolysis on bone remodeling *in vivo*. Mice rendered anemic by bleeding were also included to determine the effects associated to a short-term non-hemolytic stress. *Plasmodium* parasites replicate in RBC and provoke a potent inflammatory response which contributes to oxidative damage of infected and non-infected RBC and hemolysis [29], [30]. Albeit the anemia induced, generation of new RBC in the bone marrow is impaired in malaria due to bone marrow dysfunction [30], [31], [32] and as for β-hemoglobinopathies, high concentrations of cell-free hemoglobin are reported [33]. PHZ is used to experimentally induce anemia in animals, its mechanism of action relying on RBC lipid peroxidation (reviewed in [34]). As in malaria, robust hemolysis, high concentrations of free heme and oxidative damage are induced in PHZ-treated animals [35], [36].

## Methods

### Ethics statement

The study was conducted in compliance with the regulations from the Animal Committee of the University of Quebec in Montreal (CIPA). All procedures in mice were approved by the CIPA (protocols 0210-677-0211 and 0211-R1-677-0212).

### Mice and in vivo treatments

Female BALB/c mice (Charles River) 4–6 weeks old were used in all the experiments as young growing mice are suitable in studies designed to investigate conditions which may affect peak bone mass [37]. For infection, 10^5^ *P. c. adami* DK (556 KA) parasitized RBC were injected in mice by the peritoneal route, parasitemia was measured daily in methanol fixed tail blood smears stained with a 10% Giemsa solution in PBS and mice were sacrificed at day 8 of infection, corresponding to acute infection. PHZ (Sigma Aldrich, Canada) was administered by the peritoneal route at 40 mg/Kg body weight on day 0, and two additional injections were given at 9 am and 4 pm, on day 1, after which mice were sacrificed 6 days later. To induce non hemolytic anemia, 100 µL of blood were withdrawn from the saphenous vein every other day, three times, and mice were euthanized two days after the last blood withdrawal. Immediately following each bleeding mice received an equal volume of physiologic saline solution by the intra-peritoneal route.

### Determination of hemoglobin in blood

Hemoglobin concentrations were measured in mice daily following treatment (infection, PHZ injection or bleeding) by diluting 2 µl tail-vein blood in 500 µL Drabkin's solution (Sigma Aldrich, Canada). Hemoglobin was assayed in 96-well microtiter plates (Costar, Cambridge, MA) in a volume of 100 µl by measuring the absorption at 540 nm in a microplate reader. Values were converted to g/dL using a standard curve of human hemoglobin (Sigma Aldrich, Canada) prepared in Drabkin's solution. All samples were assessed in duplicate.

### Determination of reticulocytes and parasitemia in blood

Two microliters of blood were collected from the tips of the tails in 0.5 mL of PBS the day before and every day following treatment (infection, PHZ injection or bleeding). The blood cell suspensions were centrifuged and resuspended in 250 µL of a 0.025% glutaraldehyde solution for at least 18 h. Blood samples from *Plasmodium*-infected mice were further treated with 0.25% Triton X-100 for permeabilization, washed in PBS and resuspended in 50 µL PBS with 100 µg RNAse (4 mg/ml stock) at 37°C for 30 min. The cell suspensions were then stained with anti-CD71-FITC antibody (Cedarlane, Canada) and samples from *Plasmodium*-infected mice were additionally treated with propidium iodide (PI) (1 mg/mL stock) to exclude parasitized RBC from the analysis. The cells were analyzed in a FACScan (Becton Dickinson, USA). Parasitemia was measured daily in methanol fixed blood smears stained with a 10% Giemsa solution in PBS.

### Determination of reactive oxygen species in bone marrow cell suspensions

The marrow of tibia was recovered by flushing with cold DMEM. The cells were washed and resuspended in 2 mL DMEM supplemented with 10% FBS, 1% HEPES, penicillin (100 U/mL) and streptomycin (100 µg/mL) (Invitrogen). For ROS determination, bone marrow cell suspensions (10^6^ cells per tube) were prepared in PBS and stained with anti-F4/80-biotinilated antibody (Biolegend) and streptavidin-PERCP (BD Pharmingen) for 15 min prior to labeling with H_2_-DCFDA (Molecular Probe, Invitrogen, Canada) (100 µM) for 30 min at 37°C; the mean cellular fluorescence was measured in a FACScan cytofluorometer and analysed in F4-80^+^ and F4-80^−^ cells.

### Osteoclast progenitors and TRAP staining

To determine osteoclast progenitors, tibial bone marrow cell suspensions were stained with anti-F4-80-PE antibody (Biolegend) and analyzed by flow cytometry. In parallel, 2.9×10^7^ bone marrow cells were plated in 8 mL of αMEM medium supplemented with 10% FCS, 100 U/mL penicillin and 100 µg/mL Streptomycin (Invitrogen, Canada) overnight. Non-adherent cells were recovered and plated at 4×10^5^ cells/mL in medium with 25 ng/mL M-CSF and 50 ng/mL recombinant mouse RANKL (eBioscience) for 3 days, after which the media was replaced and cells were cultured for 4 additional days. The TRAP staining kit (B-Bridge International Inc) was used to determine tartrate-resistant acid phosphatase positive cells. Briefly, cell supernatants were removed and cells were washed once in PBS after which fixative (10% Formalin in neutral buffer) was added into the wells for 5 min. Wells were then washed three times in dH_2_O and chromogenic substrate was the added for 20–60 min. The wells were then washed in dH_2_O and stained with 10% Giemsa in PBS for coloration of nuclei. TRAP^+^ cells with ≥4 nuclei were estimated in each sample by contrast microscopy.

### Assessment of erythroid populations in the bone marrow

Freshly prepared bone marrow cell suspensions were treated with anti Ter119-PE and anti-CD71-FITC antibodies (Biolegend) for 30 min at 4°C and the cells were analyzed on a FACScan flow cytometer and the FloJo software. Ten thousand cells were acquired by flow cytometry and dead cells were excluded by staining with 7AAD (BD-Bioscience).

### Colony-forming unit assays

Bone marrow and spleen cell suspensions for colony forming assays were prepared in RPMI-1640 media supplemented with 10% FBS and antibiotics at 10× concentrations. RBC from spleen cell suspensions were lysed in Red blood cell Lysing buffer Hybri-Max® (Sigma Aldrich, Canada) prior to plating. Mature burst-forming unit-erythroid (BFU-E) were quantified in bone marrow and spleen cell suspensions using MethoCult® 03434 media. Briefly, 1×10^6^ cells/mL or 2×10^5^ cells/mL for spleen and bone marrow, respectively were prepared in RPMI 1640 and 0.3 mL were added to 3 mL of MethoCult® medium for duplicate cultures. The mix was vortexed, allowed to stand for 5 min and dispensed into 35 mm culture dishes (StemCell Technologies) using a 16 gauge blunt-end needle and a 3 cc syringe, 1.1 mL per dish. The cultures were incubated at 37°C, 5% CO_2_ in air and ≥95% humidity for 8 days after which BFU-E were identified and counted.

### Estimation of erythropoietin and bone markers concentrations in serum

Serum concentrations of erythropoietin (EPO), tartrate-resistant acid phosphatase isoform 5b (TRAP5b), N-terminal propeptide of type I procollagen (PINP) and C-terminal crosslinks telopeptide of type I collagen (CTX) were measured by EIA assays (IDS Inc, Fountain Hills, AZ and the Quantikine mouse/rat EPO Immunoassay R&D Systems, Inc). Serum concentrations of osteocalcin (OCN) were measured using a mouse osteocalcin EIA kit (Biomedical Technologies, Stoughton, MA, USA).

### Bone structure analysis

After euthanasia, bones were harvested and fixed for 24 h in 4% paraformaldehyde-phosphate buffered saline (PBS) solution at 4°C. Bone were then rinsed and transferred into fresh PBS. Bones were wrapped in PBS-soaked gauze and analyzed by micro-computed tomography (µCT) using an 1172c Skyscan system (Soquelec, Montreal, Canada) with the following configuration: x-ray potential 70 kV, 0.5 mm Al filter, 180° rotation at a 0.5° rotation step, 5 µm resolution with the small camera pixels. The raw data sets were reconstructed with the NRecon software (Skyscan, Aartselar, Belgium). The trabecular region of interest was manually drawn 2 mm from the bottom of the growth plate for 4 mm and subsequently analyzed with the CTAn software (Skyscan, Aartselar, Belgium).

### Bone histochemistry

The tibia were dissected free of soft tissue, fixed for 16 h in 4% paraformaldehyde (PF) at 4°C and rinsed in PBS. The femora were embedded in a mixture of polymethylmethacrylate (PMMA) as described by Erben [38].Two µm sections were cut on a modified Leica RM 2155 rotary microtome (Leica Microsystems, Richmond Hill, Ontario, Canada) and stained with 5% silver nitrate for 30 min under UV light for 1 h to identify mineral (Von Kossa staining). Staining for TRAP activity was carried out at 37°C in a Coplin jar placed in a waterbath as described previously [39].

### Determination of heme concentrations in serum

Concentrations of heme in serum were determined by the pyridine hemochrome method described by Falk [40]. Briefly, a stock solution of 50 mM NaOH and 20% (by volume) pyridine and 3 µL of 0.1 M K_3_Fe(CN)_6_ was placed in a cuvette and ¼ volume of serum was added and thoroughly mixed. The oxidized spectrum was then recorded and sodium dithionite (2–5 mg) was added; the spectrum of the reduced pyridine heme was immediately recorded. Heme concentrations were determined from absorbance intensity at 556 nm based on a pyridine hemochrome calibration curve determined from hemin.

### Statistical analysis

Statistical analysis was performed with a one–way ANOVA and Tukey's multiple tests, and Pearson correlation coefficients were obtained using the Prism 5 software (GraphPad, USA). *P*<0.05 was considered significant.

## Results

### Bone marrow erythropoiesis in non-hemolytic and hemolytic mice

**Figure 1:**
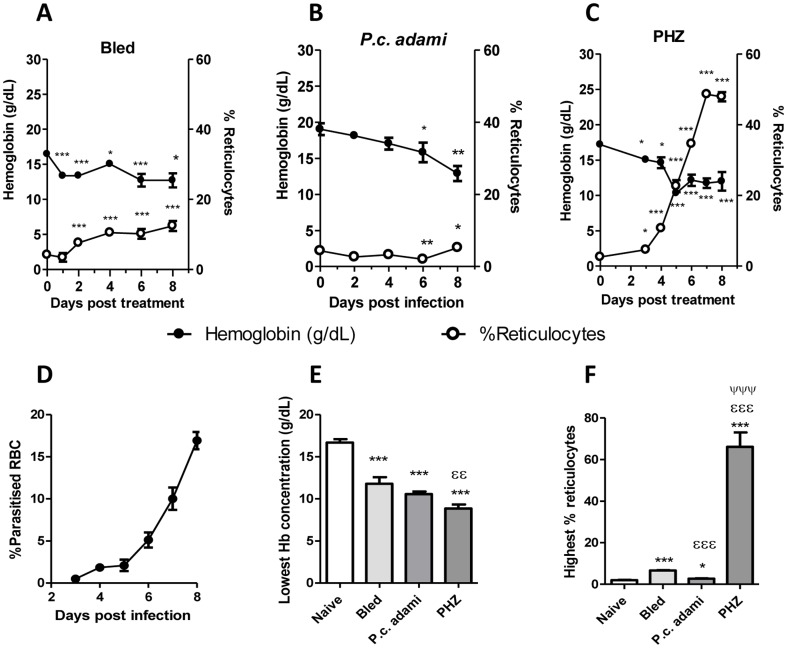
Erythroid parameters. (A–C) Blood was recovered from naive, bled, *P.c. adami-*infected and PHZ-treated female BALB/c mice prior to and every day following treatment for assessment of hemoglobin (Hb) and percentages of reticulocytes (CD71^+^). (D) Kinetics of parasitemia in *P. c. adami* BALB/c mice. (E) The lowest Hb concentrations (g/dL) and (F) highest reticulocytosis measured in mice are represented. Data are mean ± SEM from 4–7 mice per group, compiled and compared using a one–way ANOVA and Tukey's multiple test. *P<0.05, **P<0.01, ***P<0.001 are comparisons to naive; ^εε^ *P*<0.01, ^εεε^ *P*<0.001 are comparisons to bled; ^ψ^ *P*<0.05, ^ψψψ^ *P*<0.001 are comparisons between PHZ-treated and *Plasmodium-*infected mice.

Hematologic parameters and erythropoiesis were compared in bled, *Plasmodium-*infected and PHZ-treated mice. Normal hemoglobin concentrations in female BALB/c mice are 16.55±2.15 and reticulocytes represent 1–3% of total RBC in circulation [41]. Low hemoglobin concentrations occurred in all the treated mice. In bled mice, the concentrations of hemoglobin dropped significantly a day after the first blood withdrawal, remaining relatively lower during the week of experimentation and being associated with progressive reticulocytosis (Fig. 1A). A slower decline in hemoglobin concentration was noticed in *Plasmodium*-infected mice (Fig. 1B), which only became significant at day 6 post-infection and achieved its maximal level at day 8, corresponding to the moment of peak parasitemia (Fig. 1D). In respect to the day of infection, the percentages of reticulocytes were significantly lower in mice with malaria at 6 post-infection, and reticulocytosis was modestly enhanced at day 8 post-infection (Fig. 1B). In mice injected with PHZ, drastic drops in hemoglobin levels were measured, which became maximal the third day after the third PHZ injection (Fig. 1C). The drops in hemoglobin levels were accompanied by a rapid erythropoietic response, characterized by robust reticulocytosis, which was almost exponential and reached its highest levels at day 7 (Fig. 1C). No complete recovery in hemoglobin levels was noticed in PHZ-treated mice albeit the enhanced reticulocytosis measured. In summary, anemia was rapidly induced in bled and PHZ-treated, whereas in mice with malaria, significant drops in hemoglobin concentrations were only measured at day 6 post-infection, corresponding to the time when average parasitemia was ≈5% (Fig. 1D).

Significant drops in hemoglobin concentrations occurred in the three groups of treated mice (Fig. 1E), whereas reticulocytosis was significantly enhanced in PHZ-treated mice and suppressed in mice with malaria relative to bled mice (Fig. 1F). The most pronounced erythropoietic response occurred in PHZ-treated mice, which also displayed more marked reticulocytosis. At day 8, EPO levels were significantly high in mice with acute malaria relative to naive mice, lower in PHZ-treated mice compared to bled mice and comparable to controls in bled mice (Fig. 1G).

### Reduced bone mass in hemolytic anemia

**Figure 2:**
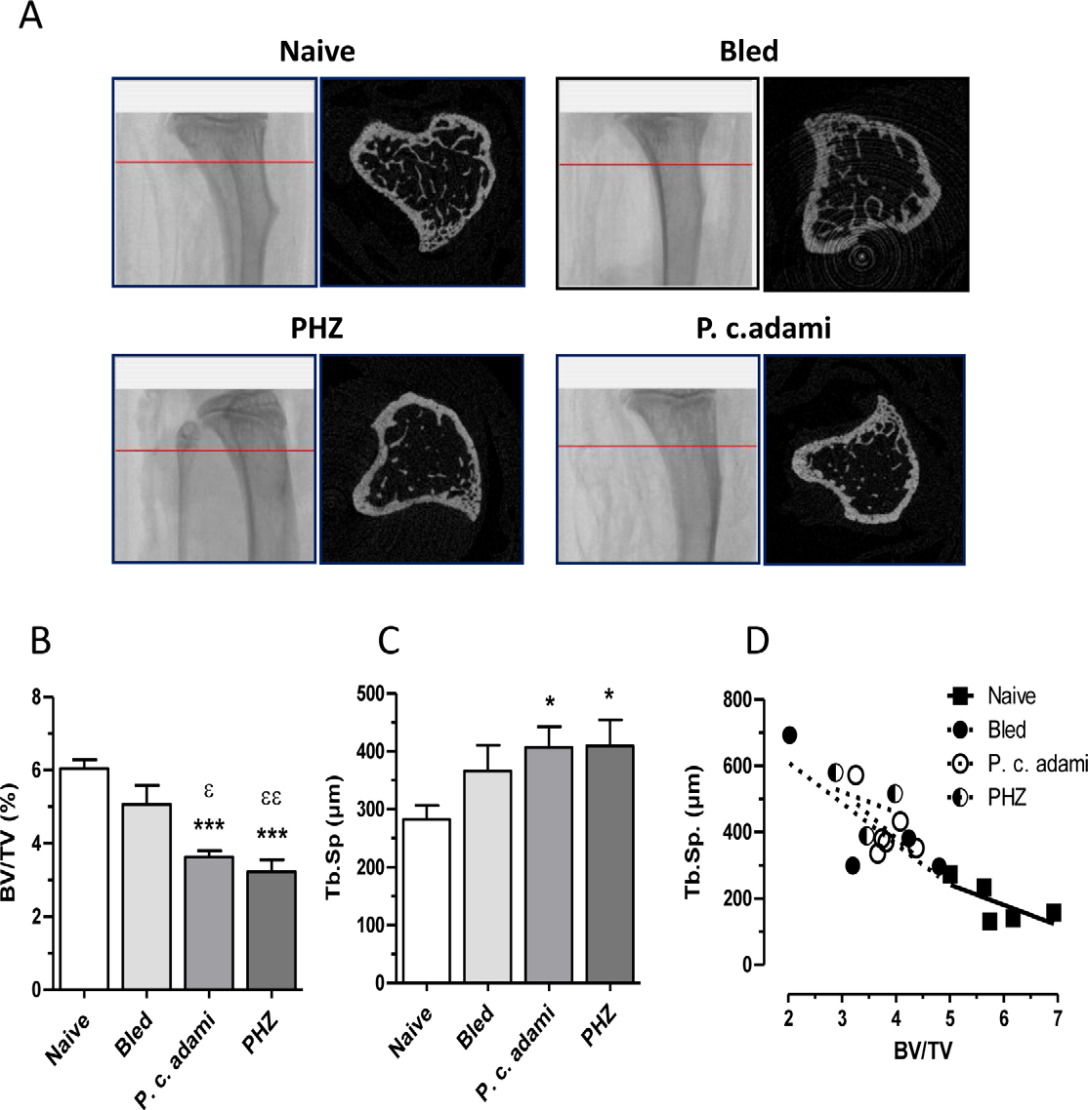
Bone architecture parameters. Naive, bled, P.c. *adami-*infected and PHZ-treated female BALB/c mice (4–6 weeks old) were euthanized and the trabecular portions of tibia were scanned. (A) Representative scan from the trabecular portion of tibia. (B–D) Ratio of bone volume (BV) on total volume (TV), trabecular spacing (Tb.Sp) were compared between each groups and Tb.Sp were plotted against BV/TV. Results are mean ± SEM from two independent experiments (n = 5–6 mice per group) compiled and compared to control mice using a one–way ANOVA and Tukey's mutiple comparison test. *P<0.05, ***P<0.001 compared to naive and ^ε^ *P*<0.05, ^εε^ *P*<0.01 compared to bled.

**Figure 3:**
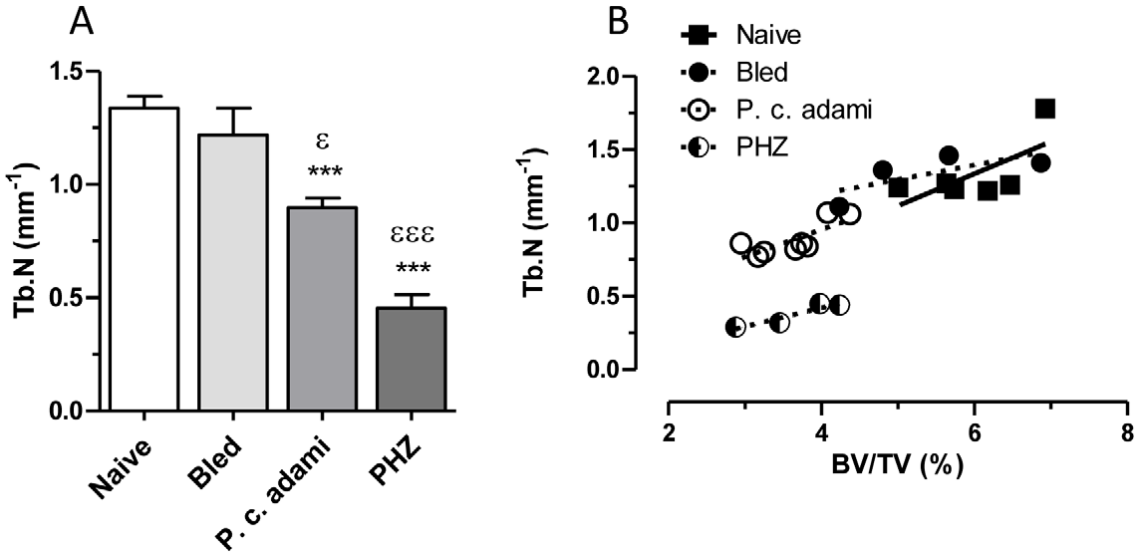
Bone architecture parameters. Naive, bled, *P.c. adami-*infected and PHZ-treated (n = 5–6) female BALB/c mice (4–6 weeks old) were euthanized and the trabecular portion of tibias were scanned. A) The trabecular number (Tb.N) was compared between each groups and plotted against the BV/TV. Results are mean ± SEM from two independent experiments (n = 5–6 mice per group) compiled and compared to control mice using a one –way ANOVA and Tukey's mutiple comparison test. ***P<0.001 compared to naive and ^ε^ *P*<0.05, ^εεε^ *P*<0.001 compared to bled.

MicroCT morphometric analysis of the tibia revealed osteoporotic features in *Plasmodium*-infected and PHZ-treated mice with HA (Fig. 2A). Quantitative morphometric values were determined for the trabecular portion of the tibiae and revealed significant reductions of specific trabecular bone volume (BV/TV) (Fig. 2B) and increased trabecular spacing (Tb.Sp) (Fig. 2C). Plotting Tb.Sp against BV/TV gave significant Pearson correlation coefficient (r^2^ = 0.77, P<0.0001) (Fig. 2D). As shown in Fig. 3A, the observed low bone mass for mice with HA associated with reduced trabecular number (Tb.N), with a significant Pearson correlation coefficient (r^2^ = 0.65, P<0.0001) (Fig. 3B). No difference was noticed for trabecular thickness (data not shown) and no significant difference was observed for the femurs.

### Analysis of erythroid precursors in non-hemolytic and hemolytic anemia

**Figure 4:**
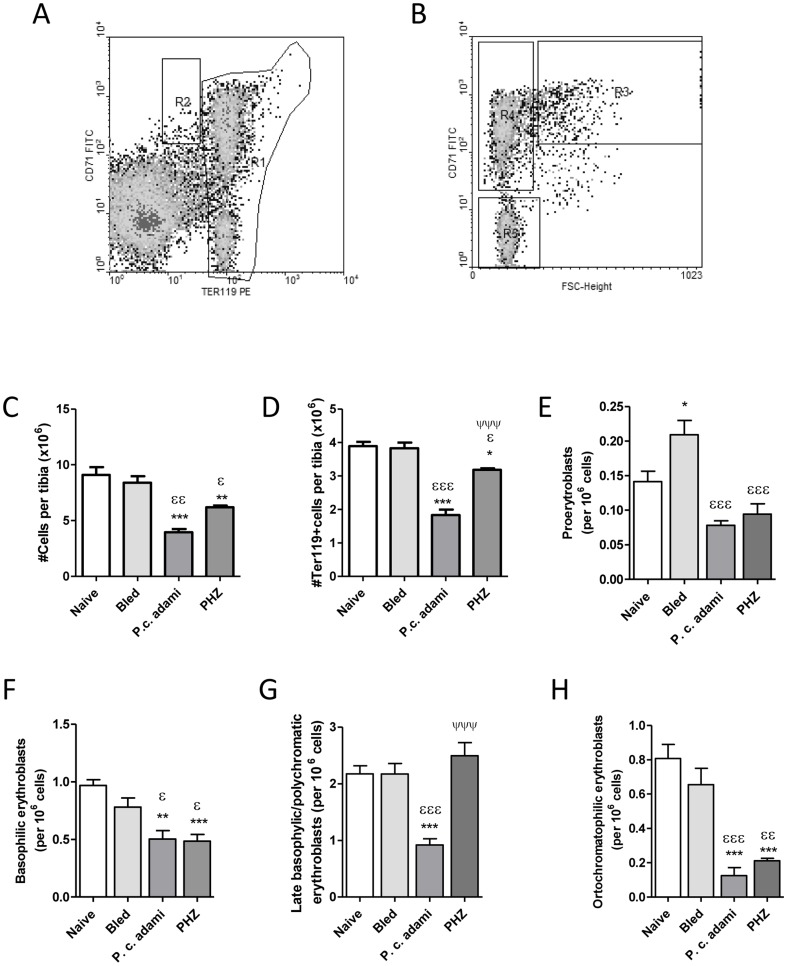
Bone marrow erythroblast subsets. Fresh bone marrow cell suspensions from the tibia of naive, bled, *P.c. adami*-infected and PHZ-treated female BALB/c mice (4–6 weeks old) were stained with anti-CD71-FITC and anti-Ter119-PE antibody for analysis of stage specific erythroblasts. Ter119^+^ cells were gated in R1 whereas a R2 region selected proerythroblasts (A). Cells within the R1 gate (Ter119 high) were further analyzed with respect to their CD71 and forward scatter (FSC) profiles (B) and the percentages of proerythroblasts (C), basophilic erythroblasts (R3) (D), basophilic/polychromatic erythroblasts (R4) (E) and late polychromatophilic erythroblasts (R5) (F) were estimated. Data are mean ± SEM from n = 4–5 mice per group and are compared to naive mice (*P<*0.05,* **P<0.01, ***P<0.001), bled mice (^ε^ *P*<0.01, ^εεP^<0.01, ^εεεP^<0.001) or between *Plasmodium-*infected and PHZ-treated mice (^ΨΨΨ^ *P*<0.001) using a one– way ANOVA and Tukey's multiple test.

Considering the reduced BMD found in the tibiae of *Plasmodium*-infected and PHZ-treated mice, the cellular status and populations of erythroid precursors in the bone marrow were investigated by flow cytometry (Fig. 4A, B). Lower cell numbers (Fig. 4C), as well as significant drops in the numbers of erythroid Ter119 positive cells (Fig. 4D) were found in mice with HA. Analysis of the subpopulations of bone marrow erythroblasts as a function of Ter119 and CD71 expression and cell size (FSC) revealed significantly higher numbers of proerythroblasts in bled mice (Fig. 4E), whereas the numbers of proerythroblasts and basophilic erythroblasts decreased significantly in *Plasmodium*-infected and PHZ-treated mice (Fig. 4F). Numbers of late basophilic/polychromatic erythroblasts were low in mice with malaria compared to control and PHZ-treated mice (Fig. 4G), whereas lower numbers of late orthochromatophilic erythroblasts/reticulocytes were measured in *Plasmodium*-infected and PHZ-treated mice (Fig. 4H).

**Figure 5:**
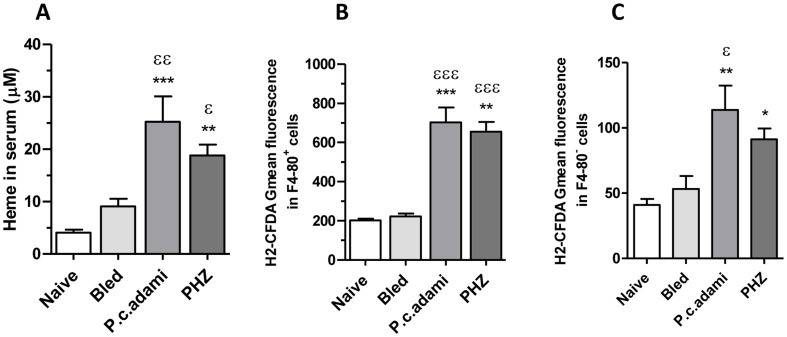
Reactive oxygen species in the bone marrow and serum heme levels. Naive, bled, *P.c. adami-*infected and PHZ-treated female BALB/c mice (4–6 weeks old) were euthanized. (A) Serum levels of heme were assessed as described in Material and Methods. (B) Bone marrow cells were recovered from the tibia by flushing and cell aliquots were stained with anti-F4-80 biotin/strepatavidin PERCP and then labelled with 2′,7′ dichlorodihydrofluorescein diacetate (H_2_-DCFDA). The mean cellular fluorescence was measured and analyzed in F4-80+ cells (B) and F4-80− cells (C) in a FACScan cytofluorometer. Results are mean ± SEM from 4 mice per group and are compared using a one–way ANOVA and Tukey's multiple test. *P<0.05, **P<0.01 and ***P<0.001 are comparisons to naive; ^ε^P<0.05, ^εε^P<0.01 and ^εεε^P<0.001 are comparisons to bled mice.

Acute hemolysis is accompanied by a rapid saturation of hemoglobin/heme scavenging and concomitant accumulation of free heme [42], [43]. Accordingly, increased concentrations of heme were measured in the serum of *Plasmodium*-infected and PHZ-treated mice the day of euthanasia (Fig. 5A). Significantly high ROS levels were also measured in F4-80^+^ (myeloid) and F4-80^−^ cells from freshly prepared bone marrow cells of mice with acute HA (Fig. 5B,C), indicating oxidative stress in the bone marrow of PHZ-treated and *Plasmodium*-infected mice.

**Figure 6:**
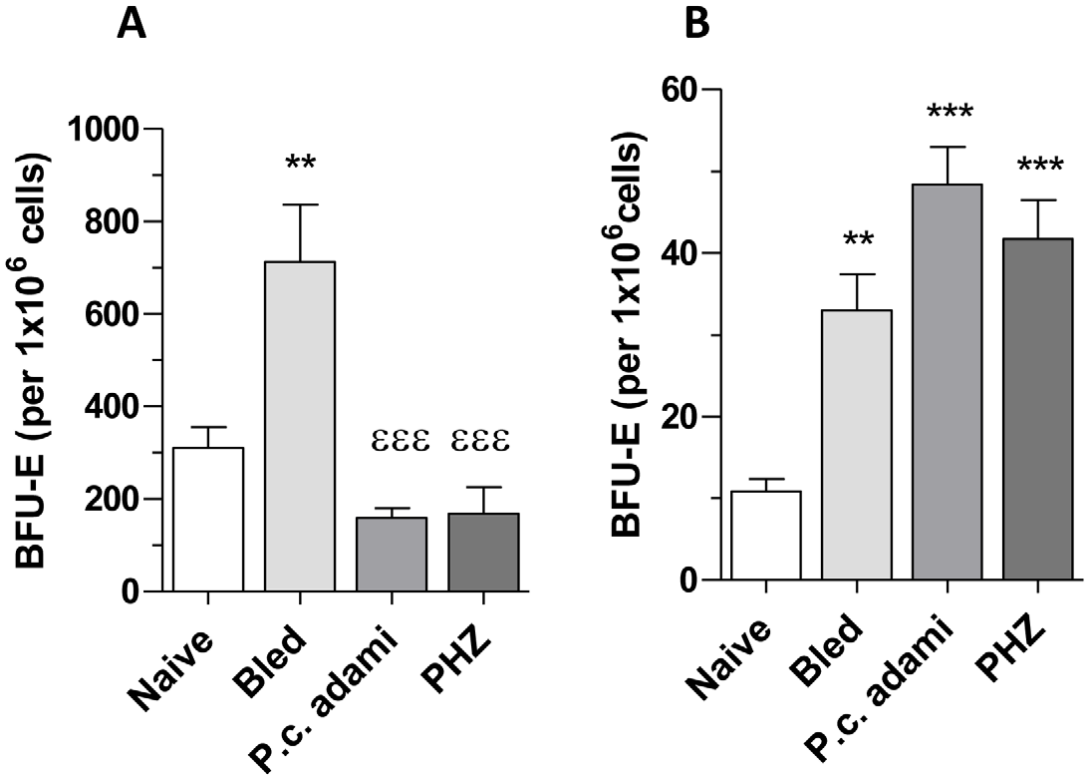
Erythroid blast forming units (BFU-E) in tibial bone marrow and spleen of mice with anemia. Naive, bled, *P.c. adami*-infected and PHZ-treated female BALB/c mice (4–6 weeks old) were euthanized and BFU-Es were determined in 12-days bone marrow (A) and spleen (B) cultures using MethoCult® 03434 media. Data are mean ± SEM from 4 mice per group and compared to naive (**P<0.001; ***P<0.01) or to bled mice (^εεε^ *P*<0.001) using a one–way ANOVA and Tukey's multiple test.

In parallel to the analysis of phenotypic markers of erythroid precursors in the tibia bone marrow, functional assays were performed to determine the capacity of bone marrow erythroid precursors to respond to EPO. Fewer bone marrow-derived BFU-E colonies were generated in bone marrow cultures from PHZ-treated and *Plasmodium*-infected mice when compared to bled mice (Fig. 6A). In contrast, significantly higher numbers of BFU-E generated from bone marrow cell cultures of bled mice when compared to naive mice (Fig. 6A). Considering that the spleen represents the major site for production of RBC in response to stress erythropoiesis in mice, BFU-Es from splenic precursors were also investigated. As shown in Fig. 6B, higher and comparable numbers of BFU-E differentiated from spleen precursors in all the anemic mice.

### Levels of bone remodeling markers and osteoclastogenesis in non-hemolytic and hemolytic mice

**Figure 7:**
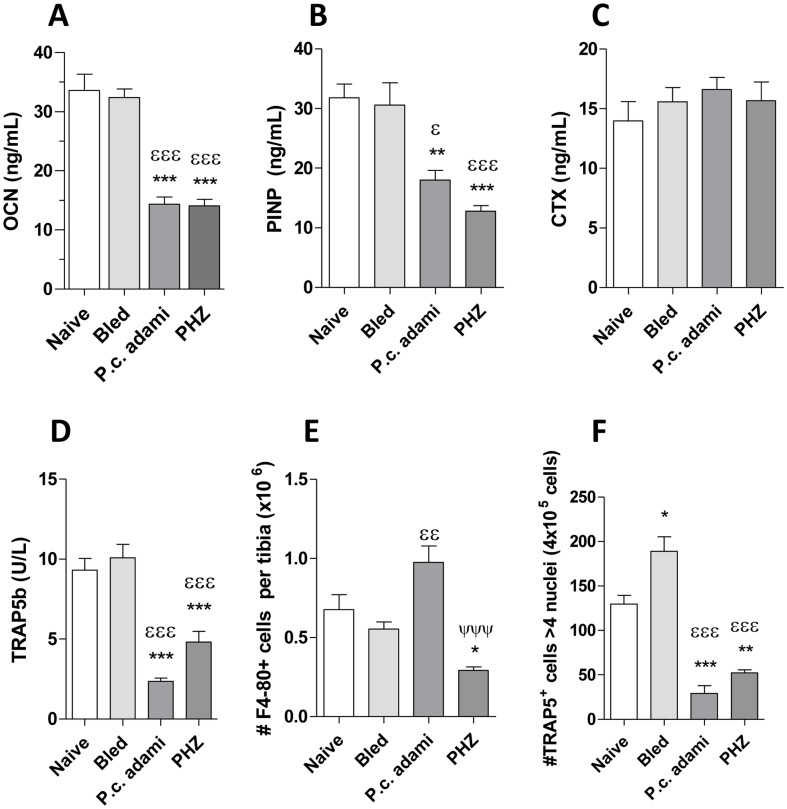
Bone remodeling markers and osteoclastogenesis in anemic mice. Naive, bled, *P.c. adami*-infected and PHZ-treated female BALB/c mice (4–6 weeks old) were euthanized eight days after treatment. Sera were assessed for concentrations of (A) osteocalcin (OCN), (B) procollagen type I N-terminal propeptide (PINP), (C) carboxy-terminal collagen crosslinks (CTX) and (D) Tartrate-resistant acid phosphatase (TRAP) 5b. Bone marrow cells were recovered from the tibia by flushing, an aliquot of each bone marrow cell suspension was stained with anti F4-80-PE antibody for estimation of osteoclast F4-80^+^ progenitors by flow cytometry (E). The remaining cells were allowed to adhere overnight after which non adherent cells were stimulated with recombinant M-CSF and RANK-L for 6 days for induction of osteoclastogenesis, and the numbers of multinucleated ostoclasts (>4 nuclei, TRAP+ cells) was determined by TRAP/Giemsa staining and confocal microscopy (D). Results are mean ±SEM from 4–10 mice per group, compared by a one –way ANOVA and a Tukey's mutiple comparison test. *P<0.05, **P<0.01, ***P<0.001 are comparisons to naïve mice; ^ε^ *P*<0.05, ^εε^ *P*<0.01, ^εεε^ *P*<0.001 are comparisons to bled mice; ^ΨΨΨ^ *P*<0.001 represents comparison between PHZ-treated and *Plasmodium*-infected mice.

To further characterize the impact of non-hemolytic and hemolytic erythropoietic stress in bone remodeling, markers for bone resorption and formation were studied. Concentrations of OCN (Fig. 7A) and PINP (Fig. 7B), indicatives of bone formation, were assessed in the serum of control and anemic mice, revealing drops in *Plasmodium*-infected and PHZ-treated mice when compared to naive mice or bled mice. Interestingly, although CTX levels, indicatives of osteoclast activity [44] remained comparable in all the mice (Fig. 7C), the concentrations of TRAP5b, which correlate to osteoclast numbers [44] were significantly lower in mice with HA (Fig. 7D), remaining comparable in bled and naive mice. Considering the drops in serum TRAP5b concentrations in mice with acute HA, we investigated the capacity of precursors to differentiate into osteoclasts *in vitro*. Higher numbers of F4-80 positive cells, which include monocyte/macrophage and osteoclast precursors were found in *Plasmodium*-infected mice compared to bled mice, whereas their numbers dropped drastically in PHZ-treated mice when compared to control or infected mice (Fig. 7E). Interestingly, the differentiation of myeloid precursors into multinucleated osteoclasts in response to RANKL and M-CSF was significantly impaired in *Plasmodium*-infected and PHZ-treated mice whereas it was enhanced in bled mice (Fig. 7F).

**Figure 8:**
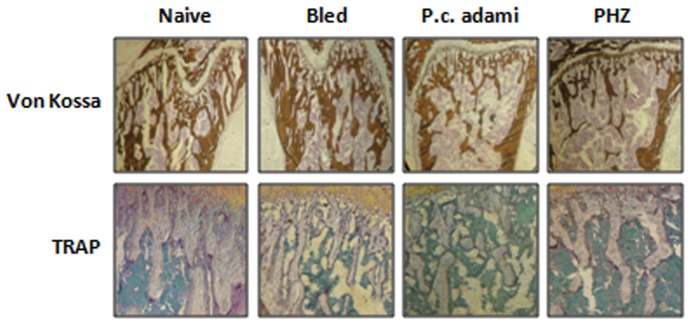
Bone histochemical analysis of tibia. Naive, bled, *P.c. adami-*infected and PHZ-treated female BALB/c mice (4–6 weeks old) were euthanized and tibias were used for Von Kossa and tartrate-resistant acid phosphatase (TRAP) staining on representative bone sections.

Von Kossa staining further confirmed the results from tibia bone scans, as comparable mineralized bone was evident in naïve and bled mice, whereas it was relatively reduced in tibias from *Plasmodium*-infected and PHZ-treated mice (Fig. 8). No major differences were observed for TRAP staining of bone tissue sections from naive and bled mice, whereas relatively weaker intensity of TRAP staining was noticed in samples from *Plasmodium*-infected and PHZ-treated mice (Fig. 8) which likely correspond to decreased trabecular number.

## Discussion

Low bone mass and high risk of fractures identified as osteoporosis are frequent in hemoglobinopathies characterized by anemia and hemolysis, which also generate dysfunctional marrow erythropoiesis and extramedular erythropoiesis. Although the sustained erythropoietic stress caused by chronic hypoxia may at long-term alter bone metabolism in affected people, the contribution for the hemolytic nature of this stress has not been yet studied. We investigated bone metabolism and erythropoiesis in mice with non-hemolytic anemia induced by bleeding and in mice with HA concurrent to infection with *Plasmodium c. adami* or PHZ treatment. Our analysis reveals that acute hemolytic stress results in significant loss in BMD, characterized by lower trabecular numbers and less mineralized bone, and important drops in the bone formation markers OCN and PINP, effects which are absent in bled mice. Our data also indicates that short-term hemolysis decreases the numbers of osteoclasts progenitors in the bone marrow and/or their ability to fuse and form multinucleated osteoclasts in response to M-CSF and RANKL, whereas bleeding enhances the ability of precursors to form osteoclasts. The latter result is in agreement with Kollet et al, which reported increased numbers of endosteal TRAP^+^ osteoclasts in mice 7 days after bleeding through a comparable procedure [45].

The loss in tibia trabecular bone mass in mice with HA was accompanied by significant drops in the numbers of bone marrow cells as well as in early and late erythroid progenitors. The drops in total eythroid precursors do not seem consequent to fewer bone marrow cells but rather to impaired proliferation. Indeed, although the percentages of immature erythroid precursors (Ter119^+^CD71^+^ cells) were similar in naive, bled and *Plasmodium*-infected mice and were enhanced in PHZ-treated mice (data not shown), significantly lower numbers of BFU-E generated from marrow cell cultures plated at comparable concentrations in methylcellulose culture media.

Considering the distinct natures of malarial and PHZ-induced hemolysis, we cannot rule out that in addition to heme, other factors may contribute to altered bone remodeling and marrow function. In this respect, the malarial pigment hemozoin has been identified as an important contributor to deficient bone marrow erythropoiesis in malaria [46], [47], but its effects in osteoclasts and osteoblasts has not been yet investigated.

We know that bleeding, infection and PHZ-treatment caused significant drops in hemoglobin concentrations and enhanced erythropoiesis in the spleen, which represents the major site generating new RBC in response to acute erythropoietic stress in mice [48]. As such, all three treatments led to important erythropoietic stress. Albeit inducing comparable erythropoiesis in the spleen, distinct kinetics of anemia was measured in these mice. Three times bleeding every 48 h led to moderate decreases in hemoglobin concentrations starting the day after the first blood withdrawal, which were accompanied by persisting but moderate reticulocytosis. In contrast, *Plasmodium* infection resulted in delayed induction of anemia and delayed reticulocytosis. In PHZ-treated mice, a relatively major hemolytic stress was induced, as hemoglobin concentrations dropped abruptly during the first three days following administration of the complete PHZ-dose and induced robust reticulocytosis until the day 6 post-treatment. Assessment of EPO levels in our group of mice at the day of euthanasia revealed significantly higher EPO concentrations in mice with acute malaria, and comparable levels in naive, bled and PHZ-treated mice. Considering that EPO levels inversely correlate with the erythropoietic activity (Lezon C et al., 1995) and the short half-life time of EPO (less than 3 hours) these results suggest ongoing erythropoietic stress in mice with malaria, which is accordance with the parasitemia kinetics and the delayed reticulocytosis measured. Indeed in BALB/c mice infected with *P. c. adami* DK parasites, reticulocytosis is readily enhanced at day 9 post-infection, when parasitemia is efficiently controlled (unpublished data). As comparable effects in BMD and bone remodeling markers were measured in mice with malaria and PHZ-treated mice, we suggest that drastic as well as relatively progressive hemolysis lead to comparable effects in the bone marrow and bone tissue. The levels of the hemoglobin and heme scavengers haptoglobin and hemopexin drastically drop in malaria [30], thalassemia and sickle cell disease [43] which confirm accumulation of free heme in all these conditions albeit their distinct kinetics.

Inefficient erythropoiesis concurrent to bone marrow dysfunction has been reported in *P. falciparum* [32], and experimental mouse malaria [30]. In *B*-thalassemia, decreased differentiation of erythroid precursors contributes to dyserythropoiesis [5], and paraspinal erythropoiesis in thalassemia intermedia also suggest insufficient bone marrow function [12]. The hemolysis in *Plasmodium*-infected and PHZ-treated mice generated high concentrations of free heme and high ROS levels in the bone marrow, both in F4-80^+^ and F4-80^−^ cells. Altogether, these data suggest dyserythropoiesis and altered bone remodeling in acute hemolytic conditions and indicate that the spleen (and probably the liver) is in charge of generating new erythrocytes. Comparatively, non hemolytic acute erythropoietic stress enhances erythropoiesis in the bone marrow and spleen but does not promote major changes in BMD and increases osteoclastogenesis *ex-vivo*.

Singbrandt et al (2011) recently reported increased trabecular spacing and lower trabecular numbers in the tibia of mice treated with recombinant EPO or PHZ [50]. Selective alteration in tibia morphology has also been reported in β^IVSII-654^ knocking thalassemic mice, which do not produce β-globin and suffer from chronic HA [51]. In the latter, the changes in bone mass seem concurrent to impaired osteoblast function and concomitant increase in osteoclast resorption activities, as drops in osteoblast surface and mineral apposition rate and increased osteoclast surface and eroded surface are measured [51], which agree with our own data. It seems feasible that high EPO concentrations in β^IVSII-654^ knocking thalassemic mice contribute to bone loss through a mechanism relying on osteoclast activation, as reported by Singbrandt and collaborators. However, although this team reported enhanced EPO levels, reticulocytosis and increased trabecular spacing in PHZ-treated mice [50], the capacity of bone marrow precursors to generate BFU-E or the effect of bisphosphonate were not investigated. Furthermore, the authors concluded, based on their finding that bisphosphonate abolished the erythropoietic response to EPO, that EPO-dependent activation of osteoclasts is required for sustainment of the erythropoietic response. However, it is important to keep in mind that amine-bisphosphonates, as those used by Singbrandt et al (2011) deplete central macrophages and may as such inhibit erythropoiesis through an osteoclast-independent mechanism [52], [53]. Based on our data, we speculate that the impact of EPO on bone resorption may be relevant in stresses as bleeding. In conditions of hemolysis, heme may impair both osteoblast/osteoclast function, and a relatively more pronounced inhibitory effect on bone formation may account for lower trabecular bone mass. Hemolysis results in high concentrations of free heme and oxidative stress, which may contribute to altered bone physiology in hemoglobinopathies and may further enhance anemia and concomitant production of EPO by inducing erythropoietic distress. Accordingly, our data with the *P.c. adami* model indicates delayed recovery from malarial anemia in mice preconditioned with heme (manuscript in preparation). Heme inhibits osteoclastogenesis *in vitro* and LPS-driven inflammatory bone loss *in vivo* through a mechanism involving HO-1 [28], and a protective role for heme/HO-1 through prevention of osteoclast activation in hemolytic/inflammatory conditions cannot be ruled out. The effect of heme on bone forming osteoblasts is less understood as induction of HO-1 has been associated to impaired maturation of osteoblasts in one study [54], whereas genetic over-expression of HO-1 increased human osteoblast stem cell differentiation in another [27].

We propose that the loss in BMD in malarial and PHZ-induced HA is concurrent to impaired bone formation and to an imbalance favoring bone resorption, as has been proposed for murine and human thalassemia [51], [55]. Since our models represent conditions of acute rather than chronic hemolytic stress, our results also suggest that hemolysis provokes rapid effects in the bone marrow and bones. Robust release of hemoglobin/heme is a major contributor to the systemic inflammatory/oxidative status in malaria and other hemolytic disorders, and inflammation is a complex response that may also affect bone tissue and exert an additional hematopoietic demand in the bone marrow. We are currently investigating the specific interactions of heme with erythropoietic niches and bone remodeling cells in absence of erythropoietic stress, which will facilitate understanding of complex pathologies as malaria and genetic hemolytic disorders.
